# The Effect of Physician Communication on Inpatient Satisfaction

**DOI:** 10.3390/healthcare10030463

**Published:** 2022-03-01

**Authors:** Massoud Moslehpour, Anita Shalehah, Ferry Fadzlul Rahman, Kuan-Han Lin

**Affiliations:** 1 Department of Business Administration, College of Management, Asia University, No. 500, Liufeng Road, Wufeng District, Taichung City 41354, Taiwan; writetodrm@gmail.com (M.M.); as350@umkt.ac.id (A.S.); 2 Department of Management, California State University, San Bernardino, CA 92407, USA; 3 International Relations Department, Faculty of Economy, Bussines and Politics, Universitas Muhammadiyah Kalimantan Timur, Jl. Ir. H. Juanda No. 15, Samarinda 75124, Kalimantan Timur, Indonesia; 4 Department of Public Health, College of Public Health, Universitas Muhammadiyah Kalimantan Timur, Jl. Ir. H. Juanda No. 15, Samarinda 75124, Kalimantan Timur, Indonesia; ffr607@umkt.ac.id; 5 Department of Healthcare Administration, College of Medical and Health Sciences, Asia University, No. 500, Liufeng Road, Wufeng District, Taichung City 41354, Taiwan

**Keywords:** inpatient, physician communication, public hospitals, satisfaction

## Abstract

(1) Background: The importance of physician-patient communication and its effect on patient satisfaction has become a hot topic and has been studied from various aspects in recent years. However, there is a lack of systematic reviews to integrate recent research findings into patient satisfaction studies with physician communication. Therefore, this study aims to systematically examine physician communication’s effect on patient satisfaction in public hospitals. (2) Methods: Using a keywords search, data was collected from five databases for the papers published until October 2021. Original studies, observational studies, intervention studies, cross-sectional studies, cohort studies, experimental studies, and qualitative studies published in English, peer-reviewed research, and inpatients who communicated with the physician in a hospital met the inclusion criteria. (3) Results: Overall, 11 studies met the inclusion criteria from the 4810 articles found in the database. Physicians and organizations can influence two determinants of inpatient satisfaction in physician communication. Determinants of patient satisfaction that physicians influence consist of amounts of time spent with the patient, verbal and nonverbal indirect interpersonal communication, and understanding the demands of patients. The organization can improve patient satisfaction with physician communication by the organization’s availability of interpreter service and physician workload. Physicians’ communication with inpatients can affect patient satisfaction with hospital services. (4) Conclusions: To improve patient satisfaction with physician communication, physicians and organizational determinants must be considered.

## 1. Introduction

Patient satisfaction is becoming a hot topic in global health policy. It is a crucial component of pay-for-performance measures and a critical predictor of care quality. Patient satisfaction is crucial for ensuring how well patients do; previous research has established a link between patient outcomes and levels of satisfaction [1]. Furthermore, patient satisfaction has been connected to subsequent usage of health services, affecting both patient compliance and treatment continuity [2]. Additionally, dissatisfied patients are more inclined to file a complaint or seek redress with the establishment to ease cognitive dissonance and a poor service experience [3]. Finally, dissatisfaction leads to poor adherence to treatment regimens, missed visits, and even negative word-of-mouth, which might dissuade others from obtaining care from the system or urge them to seek it elsewhere [4,5]. 

Measuring patient satisfaction is essential in the growing push for provider accountability. In addition, patient satisfaction surveys are vital in planning and delivering high-quality health care in hospitals where patients are actively involved in their care [6]. For medical service quality management, patient satisfaction is seen as a critical factor [7]. A patient’s opinion on the quality of service is evaluated through patient satisfaction.

Patient satisfaction surveys are in high demand as hospitals cope with physician-patient conflicts [7]. Research conducted in the United States revealed that a solid physician-patient connection and effective communication between healthcare personnel and patients are critical drivers of patient satisfaction [8,9].

Communication between physicians and patients can be multifaceted with many dimensions. For instance, a physician’s communication and interpersonal skills include the capacity to obtain information to aid accurate diagnosis, counsel effectively, deliver treatment instructions, and develop sympathetic connections with patients [10,11]. These are the fundamental clinical abilities required for medical practice, with the ultimate goal of getting the best outcome and patient satisfaction, which are necessary for the effective delivery of health care [12].

Effective physician-patient communication is providing counseling to patients on unhealthy or risky habits and is a crucial communication skill that should be incorporated into all medical appointments. Understanding how people change their behavior and setting up a systematic framework for such interventions that include the five A’s (assess, advise, agree, assist, and arrange) of patient counseling are essential parts of physician-patient communication [13].

Effective physician-patient communication serves as a motivator, incentive, assurance source, and encouragement for the patient [14,15,16]. Additionally, effective physician-patient communication can assist patients in managing their emotions, facilitating the interpretation of medical information, and allowing for a more accurate assessment of their needs, perceptions, and expectations [17,18,19]. Physicians and patients consent regarding the nature of the treatment and the necessity for follow-up is substantially associated with recovery [20,21]. When patients and their physicians communicate effectively, they are more likely to be satisfied with their care and, in particular, to share crucial information to get an accurate diagnosis, adhere to recommendations, and stick to suggested therapies [1].

The current literature has revealed that communication plays critical role in society. For many years, it has been widely recognized that communication problems between patients and physicians can be the source of impediments to good health care delivery rather than technical errors in medical care. Furthermore, high-quality physician-patient communication has improved health outcomes [11,22]. 

Nevertheless, one of the numerous hurdles to communication is health literacy. Poor health literacy makes it difficult for patients to grasp written medical information, communicate with healthcare providers, and follow self-care guidelines [23]. Unfortunately, only 12 percent of adults are proficient in health literacy [24]. 

The importance of physician communication cannot be overstated because of its impact on patient outcomes and its influence on overall hospital patient satisfaction ratings [25,26]. This component of treatment has a significant impact on a hospital’s bottom line because of patients’ impressions of their inpatient experience and their overall assessment. In addition, physician communication is one of the critical factors influencing patient perceptions of care quality [27]. Nevertheless, a physician-patient interaction is fraught with obstacles to effective communication.

There have been various systematic reviews of the relationship between communication and patient satisfaction. The studies reported that communication style [28], communication that values patient autonomy [29], nonverbal communication between patients and physicians during clinical interactions [30], and cancer care patients benefit from communication skills training [31] are factors that are associated with patient satisfaction. Despite numerous systematic reviews, no study has examined inpatient satisfaction with physician communication in a hospital setting. Therefore, this study aims to investigate the effect of physician communication on inpatient satisfaction.

## 2. Data and Methodology

### 2.1. Data

The Preferred Reporting Elements for Systematic Reviews and Meta-Analyzes (PRISMA) criteria were employed to conduct a systematic review [32]. This method has the advantage of allowing the summary and analysis of details from prior studies that are relevant to the study objective. Furthermore, using the Prisma statement, authors can perform more systematic evaluations. 

### 2.2. Search Strategy

Data was collected from five electronic databases, Scopus (1996 to 2021), Jstor (1954 to 2021), Pubmed (2007 to 2021), Web of Science (2021), and Ebsco (2021). The database was collected on 8 October 2021. The listed search terms were chosen following the instructions contained in the PICO-framed research question [33]. The combination search term of keywords to attain the potential paper were: physician communication, doctor communication, inpatient hospital customer, inpatient hospital client, inpatient hospital patient, satisfaction, contentment, gratification, and complacency. The initial search allowed for an unlimited number of languages and publishing years. The full-text references were then analyzed to determine which publications were relevant to the study’s objectives.

### 2.3. Study Selection Criteria

Reports that suited the study’s objectives were screened using the inclusion and exclusion criteria. In addition, the following criteria were employed to determine which studies should be included: (1) types of studies: original study, observational study, intervention study, longitudinal, cohort studies, case-control studies, experimental studies, qualitative studies, and cross-sectional studies in the English language publication on peer-reviewed studies or papers published between 2011 and 2021; (2) types of participants included inpatients who had communicated with the physician in the public hospital; (3) types of exposure included communication between the physician and patient during hospitalization, and (4) types of outcome involved patient satisfaction levels after communicating with the physician directly. 

The exclusion criteria are as follows: (1) types of studies: studies with meta-analysis studies, systematic reviews, database studies, literature reviews, review papers, protocols, abstract only publications, or abstracts only at symposium proceeding books; (2) types of participants: patients who use clinic or primary health care and have no information about the inpatients or outpatients. Patients who did not communicate directly with physicians were excluded (via nurses or caregivers). Also, psychiatrist-patient communication encounters were excluded because their nature differs from hospital medical encounters. Articles with more outpatient populations than inpatients were excluded. Two reviewers (AS and FFR) independently evaluated the titles, abstracts, and complete research texts based on a literature search. When there was a different opinion between the two reviewers, discussions would be held until consensus was reached; the third reviewer (MM) was involved in decision making when there were disagreements between them.

### 2.4. Data Extraction and Synthesis

For each study included in the qualitative synthesis, the first reviewer (AS) retrieved pertinent data, and the second reviewer (FFR) independently verified the accuracy. The following data was gathered from the chosen studies: author, publishing year, country of study, study design, sample size, hospital ownership, outcome measurement, and satisfaction findings. As a result of conversations between the two reviewers and the assistance of a third reviewer, disagreements were resolved (MM). The Microsoft Excel program was used to record all of the data.

### 2.5. Quality Assessment

We used the Joanna Briggs Institute’s (JBI) Critical Checklist, tailored to each study’s design. JBI checklists for critical appraisal include eight items for cross-sectional studies, nine items for experimental studies, and eleven items for cohort studies. Each item on the checklist was evaluated using the terms “yes”, “no”, “unclear”, and “not applicable” [34]. Any dissent was resolved by discussion between reviewers.

### 2.6. Statistical Analysis

SPSS 23.0 (IBM Corp, Armonk, NY) was used to analyze the data. Cohen’s Kappa statistics and percentage agreement were utilized to measure the degree of agreement between two reviewers on study selection and quality rating. The Kappa result interpretation is valued ≤0 as indicating no agreement, 0.01–0.20 as none to slight, 0.21–0.40 as fair, 0.41–0.60 as moderate, 0.61–0.80 as substantial, and 0.81–1.00 as almost perfect agreement [35].

## 3. Content Analysis and Result

### 3.1. Search Result

**Figure 1:**
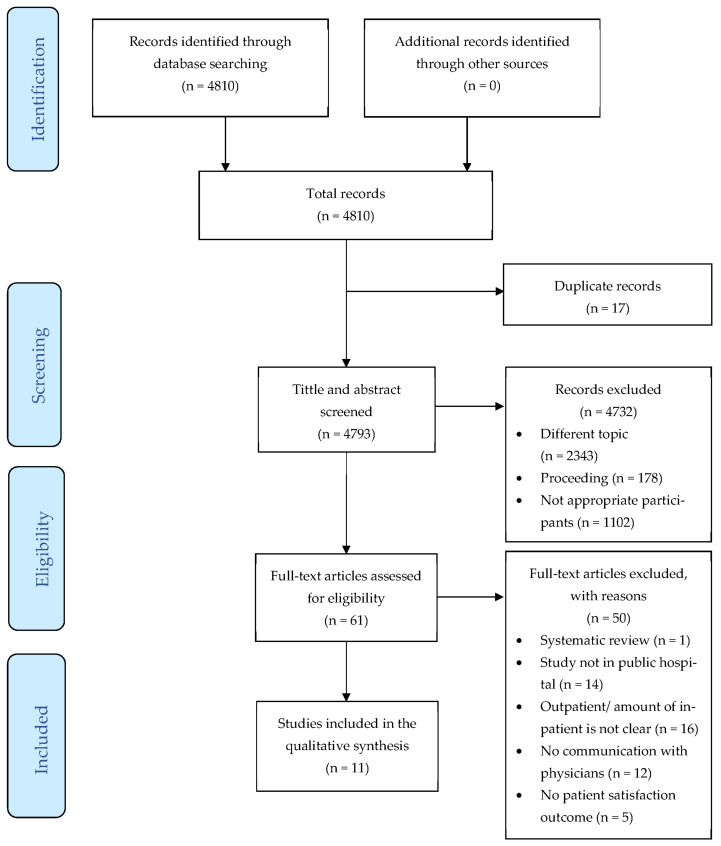
PRISMA Flow diagram.

Based on the five electronic databases, the number of articles at the initial screening was 4810. After removing the duplication, the number of articles screening the title and abstract was 4793. In the title and abstract screening stage, the researchers reviewed related papers conducted between 1954 and 2021. Finally, 11 articles that qualify as included in the study were found in the full-text eligibility stage. Figure 1 illustrates the process review in detail.

The agreement between two independent reviewers for screening the title and abstract was substantial in Table 1 (Kappa 0.666, *p*-value < 0.001), Table 2 showed full-text screening and was substantial (Kappa 0.640, *p*-value < 0.001), and Table 3 showed studies included in the qualitative synthesis and was substantial (Kappa 0.621, *p*-value 0.026).

### 3.2. Study Characteristics

The included studies are summarized in Table 4. The year of publication of included studies was between 2007 and 2021. All of the papers in this collection were written in English and published. There are differences in several research locations that were found; two studies were conducted in Ethiopia [36,37], two studies in China [38,39], one study in Hong Kong [25], one study in the USA [40], one study from Germany, Switzerland, and Austria [41], one study in Indonesia [42], one study in Iran [26], one study in Korea [43] and one study in Australia [44]. Nine articles used a cross-sectional study method, one article was an experimental study, and one article was a cohort study.

In the 11 studies that were included in this study, it was found that different types of measures were used to analyze patient satisfaction. For example, the outcome measurement employed is the one that is applicable in their country, or they developed standardized structured questionnaires for data collection after reviewing pertinent literature, or the company approved the questionnaire (Table 5).

### 3.3. Participants’ Characteristics

The total number of participants from the 11 studies was 121,689 people. One study did not identify the number of patients that participated but only offered information regarding the number of hospitals that participated in the study [40]. This study has no age limit. All patients are considered to be taking a survey to answer questions. Nine articles were conducted in public hospitals, and four in public and private hospitals. It is confirmed that the four studies conducted in public and private hospitals have a larger sample size in public hospitals than in private hospitals. Thus, it is consistent with this study’s objective, which examines public hospitals.

## 4. Discussion

### 4.1. Overall Satisfaction

This study summarizes articles related to physician-patient communication satisfaction systematically. Six articles [25,26,39,41,42,43] out of the 11 articles met the review criteria, showing either that patient satisfaction with hospital services was more than 80%, or that an increase after intervention was higher than the national survey results. Conversely, five articles [36,37,38,40,44] demonstrated that the patients were less satisfied. Patient satisfaction was investigated with three types of study designs, namely cross-sectional studies (9 articles), experimental studies (1 article), and cohort studies (1 article).

### 4.2. Determinant of Physician Communication Satisfaction

- Amounts of time spent with the patient: The study’s findings reveal that the amount of time spent with the patient has the most significant impact on patient satisfaction with physician communication. Five articles [25,36,37,38,43] show that patients expect physicians to spend more time communicating with them during interactions, especially in ward rounds. Even though there are no commonly agreed time limits for conversation or physical examination, most researchers believe that more time improves physician and patient treatment quality. Moreover, the frequency of ward rounds should be increased.
- Verbal and nonverbal indirect interpersonal communication: Three articles [36,39,42] explain that direct interpersonal communication is the key to patient satisfaction. In communicating with patients, physicians must be knowledgeable, friendly, informative, empathetic, be courteous, show respect, be open, be supportive, be positive, treat patients equally, be focused, show good behavior, have a good attitude, and feel valued. Moreover, the physician must be sensitive to the patient’s body movement and postural indicators. Additionally, physicians who were effective at expressing emotion through nonverbal communication received higher scores on the art of care from patients than physicians who were ineffective communicators. Furthermore, communication-based models tend to be more successful than communication-based on the picture at increasing patient satisfaction, reducing patient discomfort, improving communication ease, augmenting patient adherence, enhancing the interaction between physician and patient, and enhancing patient outcomes.
- Understand the demand of patients: The patient’s demands that affect physicians’ communication satisfaction are summarized from five articles [25,36,38,39,41,44] that support this argument. Several patient demands which affect patient satisfaction are expected to be obtained from physicians. For example, these include complete information about their illness; more input into their care and treatment decisions; listening to their views of treatment; receiving notification before treatment; having their dignity respected; allowing the patient’s family to speak with the physician; and notifying patients of danger signals regarding their disease/treatment/possible complications of the condition after they went home.

### 4.3. Organizational Determinants

In this study, organizational determinants also affect patients’ satisfaction with physician communication, as the findings of four articles [36,40,44] have been collected. These findings are quite surprising because two-way communication only involves physicians and patients, but the hospital can influence patient satisfaction. This study found two determinants that the hospital can improve that affect patient satisfaction with physician communication:

- Interpreter service and the simplifying of medical terms into layperson terminology: The key in physician-patient communication is understanding the language being spoken in order to provide their complaints. The study revealed that some patients could not communicate with physicians and nurses owing to language barriers [36]. Almost unanimously, they expressed dissatisfaction with the lack of interpretation services. Additionally, it was discovered in this study that the language barrier was not solely due to the patient’s inability to communicate in the same language as the physician but was also due to the physician’s inability to translate medical terminology into plain terms that were easily understood by the patient [44]. Therefore, hospital management is obligated to provide translation services using terminologies that patients easily understand.
- Physician’s workload: The study results [40] found that physician workload substantially affects patient perceptions of physician communication. This result is due to the prevalence of physician fatigue, which could impact patient quality of treatment and experience. Due to the hospital’s objective in gaining market share and aligning physician incentives, the hospital relies increasingly on full-time physicians who determine physician workload or staffing levels. In addition, hospitals with a higher profit margin and a more significant physical footprint have lower patient ratings for physician communication [40]. Moreover, hospitals seeking big profits tend to use internships or residents who are paid less but also lack communication skills [37].

## 5. Conclusions

Our study found that physician-patient communication affects patient satisfaction. Of the 11 articles in this study, all articles show a straight comparison between physician-patient satisfaction and health care providers’ satisfaction. The studies were conducted in 11 countries; Ethiopia, China, Hongkong, USA, Germany, Switzerland, Austria, Indonesia, Iran, Korea, and Australia. Nine articles employed cross-sectional studies, one article utilized experimental studies, and one article employed cohort studies.

This study revealed the determinant factors that affect patient satisfaction toward physician communication. Therefore, this study offers practical implications for both physicians and organizations. Concerning the physicians, we offer several ways to increase patient satisfaction.

First, physicians are advised to allow more time to interact with patients. This finding is supported by previous research [45], which suggests that patients are more satisfied with physicians when discussing test results and physical examination findings that exceed 15 min and are less satisfied with shorter appointment periods. Many patients complain of a short ward round time due to the high number of patients [38]. In contrast, ward rounds provide a vital chance for physicians and patients to communicate. Therefore, the frequency of ward rounds should also be increased. At least twice a day, the physician should perform ward rounds; depending on the patient’s state of health, adaptations to the physician’s directions are possible, thereby enhancing patient health and care. It helps to build knowledge of the patient’s condition and discuss the diagnosis and the treatment strategy. Patients expect physicians to stop by during rounds to check on them and see whether they can go home. Additionally, even when physicians spend about the same amount of time with each patient, patients report feeling that they are not receiving any special attention.

The second way involves verbal and nonverbal indirect interpersonal communication between physician and patient. The results of the previous systematical review of physician-patient communication in primary care offices [46] also found that physicians verbal and nonverbal behavior can increase patient satisfaction. In addition, in the study of maternal needs, it was found that verbal and non-verbal physician communication was beneficial with regards to ensuring a healthy delivery [47].

Last, physicians can understand the demands of patients. These results support the previous study on meaningful communication from the patient’s perspective [48] and the systematical review [49] regarding determinants of patient satisfaction which also comes to the same conclusions.

This study offers several recommendations for the hospital to increase patient satisfaction. First, it is recommended that hospitals provide interpreter services. The findings of this study corroborate those of recent research [45] on the impact of medical interpretation services on health care quality.

Second, the hospital must be able to control the physician’s workload. This recommendation relates to the time spent with the patient, as previously addressed. The problem of physician workload or physician staffing levels also results in less time and energy spent with patients, adversely affecting the quality of patient-physician communication. Hospital improvement is often achieved by emphasizing effectiveness and efficiency and increasing physicians workloads. However, effectiveness and efficiency may harm patients’ experiences of the quality of the patient-physician communication. Patient comfort and physician workloads are essential considerations when hospitals want to operate efficiently. Previously conducted research examined the relationships between nurse intensity, the intensity of the working environment, resources for the nursing profession, and patient satisfaction using nurse staffing measures [50].

Knowing the factors influencing patient satisfaction might help physicians, hospitals, and policy-makers develop and implement successful ways to improve healthcare services. This study shows that patients’ satisfaction levels with regard to physician communication does not only depend on improvements in the physician’s personality, but also on hospital organizational factors that affect increasing patient satisfaction. Comprehensive patient satisfaction models may help policymakers identify patient requirements, create physician and patient roles, manage demand and capacity, and achieve needed service quality.

The theoretical contributions of this study supports other published studies. In addition, the methodological concerns of the results of this study can guide directions in future research. Furthermore, this study fills in some of the gaps in systematic review studies regarding patient satisfaction with physician communication.

The study’s main drawback is the resulting bias associated with patient satisfaction with hospital and physician communication. We must admit that some of the articles found in this study did not focus on assessing patient satisfaction of physician’s communication alone but rather related to overall hospital services. Nevertheless, there were aspects of satisfaction with patient and physician communication. In this study, we have focused on patient satisfaction with physician.

Additionally, the review of multiple types of research conducted in diverse nations, hospitals, departments, and demographics and the variability of theoretical frameworks, study designs, and measurements may have contributed to the inconsistency and incomparability of findings. Although this study’s process follows the implementation of a systematic review (PRISMA), we suggest that further research is needed due to the limitations of this study. Furthermore, this study’s findings do not include COVID-19 research articles regarding patient and physician communication during the pandemic. Most studies during the pandemic use telemedicine or other new subjects, which are now globally an important means of communication between patients and health care providers [51,52] that cause indirect communication [53,54,55,56,57,58]. At the same time, this study examines direct physician-patient communication, especially physician visits at the patient’s bedside without intermediaries.

## Figures and Tables

**Table 1:** Agreement title and abstract screening percentage and kappa result between first and second reviewer.

Symmetric Measures					
		Value	Asymptotic Standard Error ^a^	Approximate T ^b^	Approximate Significance
Measure of Agreement	Kappa	0.666	0.141	48.928	0.000
N of Valid Cases		4793			

^a^ Not assuming the null hypothesis. ^b^ Using the asymptotic standard error assuming the null hypothesis.

**Table 2:** Agreement full-text screening percentage and kappa result between first and second reviewer.

Symmetric Measures					
		Value	Asymptotic Standard Error ^a^	Approximate T ^b^	Approximate Significance
Measure of Agreement	Kappa	0.640	0.192	5.039	0.000
N of Valid Cases		61			

^a^ Not assuming the null hypothesis. ^b^ Using the asymptotic standard error assuming the null hypothesis.

**Table 3:** Agreement studies included in the qualitative synthesis percentage and kappa result between first and second reviewer.

Symmetric Measures					
		Value	Asymptotic Standard Error ^a^	Approximate T ^b^	Approximate Significance
Measure of Agreement	Kappa	0.621	0.335	2.225	0.026
N of Valid Cases		11			

^a^ Not assuming the null hypothesis. ^b^ Using the asymptotic standard error assuming the null hypothesis.

**Table 4:** Study characteristics of included studies.

	Author, Year	Country	Study Design	Sample Size	Hospital Ownership	Outcome Measurement	Overall Satisfaction	Satisfaction Finding
1	Wong et al., 2011 [25]	Hongkong	cross-sectional study	1264 patients	public and private	Picker Patient Experience Questionnaire-15 (PPE-15)	satisfied	The physician-patient relationship had a substantial effect on patient satisfaction. The findings indicate that ‘desire to be more involved in decisions about care and treatment’, ‘respect for the patient’s dignity, ‘patients’ family has sufficient opportunity to speak with a physician’, and ‘tell about danger signals regarding illness/treatment after went home’ are all significant predictors of global satisfaction scores.
2	Zewdneh et al., 2011 [37]	Ethiopia	cross-sectional study	211 patients	public	Lehman’s and Kraan’s standard checklist (Maastricht checklist)	needs further improvements	The total score assessment indicated that interns performed poorly, and residents and consultants performed poorly, demonstrating an obvious lack of communication skills and behavior. All physician categories scored poorly on nearly every checklist item, indicating that adequate attention has not been paid to physicians’ communication skills and behavior. Medical training currently has little effect on the communication ability and behavior of physicians and their trainees, meaning that the problem may be pervasive in medical practice across the country due to the curriculum shortfall. The analysis of interaction time for psychosocial exchange revealed that 87 percent of encounters occurred during the intervals of 5–7 min and 8–10 min.
3	Woldeyohanes et al., 2015 [36]	Ethiopia	cross-sectional study	189 patients	public	Two sets of standardized structured questionnaires were created for data collection after conducting a literature study	needs further improvements	The vast majority (88.9 percent) of patients could converse freely with nurses and physicians. However, the remaining patients were unable to speak with nurses and physicians due to the language barrier, and almost all of them (95.2 percent) expressed dissatisfaction with the lack of translator services. Regarding the physician’s service, 60.3 percent of patients expressed satisfaction with their knowledge, courtesy, and respect for them. However, 62.4 percent of patients expressed dissatisfaction with the degree of education and communication regarding their illness, and 69.8 percent reported receiving insufficient information. The high volume of patients expecting to see a physician results in a paucity of time and a poor level of education among the patients, which may act as a barrier to communication understanding.
4	Al-Amin and Makarem, 2016 [40]	USA	cross sectional study	2756 hospitals	Public and private	Hospital Consumer Assessment of Healthcare Providers and Systems (HCAHPS)	needs further improvements	By investigating the influence of a variety of hospital factors on patient perceptions of physician communication, we can identify organizational issues that inhibit physician performance in an inpatient situation: Organizational characteristics are associated with ineffective patient-physician communication. A physician’s workload substantially affects patients’ perceptions of physician communication. Profitable institutions and hospitals with a greater patient population earn lower patient satisfaction scores for physician communication.
5	Zin et al., 2016 [41]	Germany, Switzerland, and Austria	cross-sectional study	116,325 patients	Public and private	German Inpatient Satisfaction Scale (GISS)	satisfied	The first component, dubbed satisfaction with medical physicians’ care, is as follows: ‘The medical physicians are sufficiently informed about patient care and respond to questions during their ward rounds in an informative and friendly manner’, ‘The diagnoses are conveyed with a great deal of empathy’, ‘The patient was well informed about the potential complications of the condition after leaving the hospital’, ‘The medical care has been successful thus far’, ‘The pain has been effectively alleviated’.
6	Hu et al., 2016 [39]	China	Experimental Study	240 patients	public	Demographic Information Survey Scale and a Medical Interview and Satisfaction Scale (MISS)	satisfied	In comparison to picture-based communication, model-based communication appears to be more effective at increasing patient satisfaction, alleviating patient distress, increasing communication comfort, increasing patient compliance, strengthening the physician-patient relationship, and improving patient outcomes.
7	Ke et al., 2018 [38]	China	cross-sectional study	872 patients	public	Inpatient Patient Satisfaction Questionnaire Developed by Chongqing Zhidao Hospital Management Corporation	needs further improvements	As a result of inadequate communication, complaints are primarily aimed at the two primary types of clinical care staff, physicians and nurses. Patients expressed dissatisfaction with physicians’ ward rounds. However, the ward round is a critical opportunity for clinicians and patients to communicate. In addition, ward rounds occurred only once a day. Thus, the physician should perform ward rounds at least twice daily; depending on the patient’s state, adjustments to the physician’s instructions can be made, improving the patient’s health and providing appropriate treatment. Physicians did not verbally communicate with numerous patients. As a result, patients experienced anxiety the day before surgery.
8	Effendi et al., 2019 [42]	Indonesia	cross-sectional study	72 patients	public	Openness, empathy, supportiveness, positiveness, and equality	satisfied	Openness, empathy, supportiveness, positivity, and equality all substantially affected patient satisfaction. These factors were considered during direct interpersonal communication (also known as face-to-face or direct communication) between physicians and patients.
9	Ali and Koorosh, 2019 [26]	Iran	cross-sectional study	285 patients	public	The Jefferson Scale of Patient’s Perceptions of PhysicianEmpathy (JSPPPE)	satisfied	There is a significant positive association between perceptions of physician empathy and patient satisfaction. In addition, factors such as respect for patients’ ideas and words and understanding patients’ concerns and their unique needs may have affected patient satisfaction.
10	Chae et al., 2021 [43]	Korea	cross-sectional study	2181 patients	public	The questionnaire was developed from Tools for Assessing Patient Satisfaction with Services from Hospitalists and Hospital Consumer Assessments from Healthcare Providers and Systems	satisfied	Patients treated by hospitalists report higher satisfaction because physicians respond more quickly. For example, patients can see their physician more than twice a day, meet when asked to, and meet immediately upon admission.
11	Chia and Ekladious, 2021 [44]	Australia	Cohort study	50 patients	public	a multiple-choice questionnaire was devised specifically for the study	needs further improvements	Enhancements to physician communication regarding treatment alternatives, the use of language that is easily understood by laypeople (lay terminology), and the verification of patients’ comprehension of the information provided Patients aged <65 years are less likely to feel informed about their condition or treatment than patients aged >65 years

**Table 5:** Methodological Quality of Included Studies.

Joanna Briggs Institute Checklists	Wong et al., 2011	Zewdneh et al., 2011	Woldeyohanes et al., 2015	Al-Amin and Makarem, 2016	Zin et al., 2016	Hu et al., 2016	Ke et al., 2018	Effendi et al., 2019	Ali and Koorosh, 2019	Chae et al., 2021	Chia and Ekladious, 2021
Cross-sectional studies											
Are the criteria for inclusion in the sample clearly defined?	1	1	1	1	1		1	1	1	1	
Were the study subjects and the setting described in detail?	1	1	1	0	1		1	1	1	1	
Was the exposure measured validly and reliably?	1	1	1	1	1		1	1	1	1	
Were objective, standard criteria used for measurement of the condition?	1	1	1	1	1		1	1	1	1	
Were confounding factors identified?	1	1	1	1	1		1	1	1	1	
Were strategies to deal with confounding factors stated?	1	1	1	1	1		1	1	1	1	
Were the outcomes measured validly and reliably?	1	1	1	1	1		1	1	1	1	
Was appropriate statistical analysis used?	1	1	1	1	1		1	1	1	1	
Experimental Studies											
Are ‘cause’ and ‘effect’ clear in the study (i.e., there is no confusion about which variable comes first)?						1					
Were the participants included in any similar comparisons?						1					
Were the participants included in any comparisons receiving similar treatment/care other than with regard to the exposure or intervention of interest?						1					
Was there a control group?						1					
Were there multiple measurements of the outcome, both before and after the intervention/exposure?						1					
Was follow-up complete, and if not, were differences between groups in terms of their follow-up adequately described and analyzed?						1					
Were the outcomes of participants included in any comparisons measured in the same way?						1					
Were outcomes measured reliably?						1					
Was appropriate statistical analysis used?						1					
Cohort Study											
Were the two groups similar and recruited from the same population?											1
Were the exposures measured similarly to assign people to both exposed and unexposed groups?											1
Was the exposure measured validly and reliably?											1
Were confounding factors identified?											1
Were strategies to deal with confounding factors stated?											1
Were the groups/participants free of the outcome at the start of the study (or at the moment of exposure)?											1
Were the outcomes measured validly and reliably?											1
Was the follow-up time reported and sufficient to be long enough for outcomes to occur?											1
Was follow-up complete, and if not, were the reasons for follow-up loss described and explored?											1
Were strategies to address incomplete follow-up utilized?											1
Was appropriate statistical analysis used?											1

## Data Availability

Not applicable.
